# Apoptosis, inflammatory and innate immune responses induced by infection with a novel goose astrovirus in goose embryonic kidney cells

**DOI:** 10.3389/fcimb.2024.1452158

**Published:** 2024-10-22

**Authors:** Zhanpeng Hou, Shaobing Jin, Yu Liang, Haiyue Wang, Danli Jiang, Nan Cao, Minhua Sun, Yunbo Tian, Wenjun Liu, Danning Xu, Xinliang Fu

**Affiliations:** ^1^ College of Animal Science & Technology, Zhongkai University of Agriculture and Engineering, Guangzhou, China; ^2^ Waterfowl Healthy Breeding Engineering Research Center of Guangdong, Zhongkai University of Agriculture and Engineering, Guangzhou, China; ^3^ Institute of Animal Health, Guangdong Academy of Agricultural Sciences, Guangzhou, China

**Keywords:** GoAstV, apoptosis, inflammatory response, innate immune, NLRP3

## Abstract

**Introduction:**

Since 2016, a highly lethal visceral gout induced by infection with the novel goose astrovirus (GoAstV) resulted in an ongoing outbreak in goslings in China, with a mortality rate ranging from 10% to 50%, and causing considerable economic losses in the goose industry. However, the pathogenesis of GoAstV and the molecular mechanism by which kidney lesions are induced by GoAstV infection are unclear.

**Methods:**

In the present study, a GEK cell infection model for GoAstV was established, and the apoptosis, inflammatory and innate immune responses induced by GoAstV were investigated in GEK cells.

**Results:**

The results shown that the expression of proapoptotic proteins, including Bax, caspase-3, caspase-9 and cytochrome *c*, increased in the infection group; however, the expression of the antiapoptotic protein Bcl-2 decreased, indicating that apoptosis was induced by GoAstV infection in GEK cells. Besides, the activation of the RIG-I/MDA5 pathway and the downstream upregulation of proinflammatory cytokines, including the adapter proteins MAVS, IRF7 and NF-κB and the proinflammatory cytokines IL-6, IL-8 and TNF-α, were detected in GEK cells infected with GoAstV. In addition, GoAstV infection induces the activation of the NLPR3 pathway and further stimulates the increased production of IL-1β. In summary, the present study revealed that GoAstV infection could induce apoptosis and the activation of the RIG-I/MDA5 and NLRP3 pathways in GEK cells, as well as the massive release of proinflammatory cytokines.

**Discussion:**

These results are helpful for elucidating the molecular mechanism of pathological lesions in the kidney in gout goslings infected with GoAstV and the interaction between GoAstV and the innate immune system of the host.

## 1 Introduction

In recent years, a highly lethal case of visceral gout, which is characterized by visceral urate deposition, hemorrhage, and swelling of the kidneys, has affected goslings in China, with a mortality rate ranging from 10% to 50% and causing considerable economic losses to the goose industry (Niu et al., 2018; Zhang et al., 2018a). Several studies have shown that the pathogen causing gout outbreaks in goslings is a novel goose astrovirus (GoAstV) (Yang et al., 2018; Zhang et al., 2018b). In addition, GoAstV has been reported to undergo cross-species transmission from geese to ducks and to be vertically transmitted from breeding geese to goslings (Wei et al., 2020a, b), which poses a serious threat to the goose industry. Currently, there are two different GoAstV genotypes circulating in geese, GoAstV-1 and GoAstV-2, and GoAstV-2 is the predominant genotype responsible for gout outbreaks in goslings (Fu et al., 2022; Li et al., 2021).

Kidneys from geese infected with GoAstV showed severe lesion and histopathological changes, including glomerular swelling, tubular epithelial cell shedding and necrosis, and inflammatory cell infiltration (Yang et al., 2018; Zhang et al., 2018a). Moreover, the virus titer in the kidney is the highest among the tissues and organs affected by infection (An et al., 2020; Zhang et al., 2018b); Thus, the kidney is considered to be the target tissue or target organ for GoAstV infection. In addition, kidney is responsible for uric acid excretion, and GoAstV infection can cause kidney lesions and decrease renal excretion, which contribute to hyperuricemia and gout formation (Wu et al., 2020). However, the molecular mechanism of pathological lesions in the kidney in gout gosling induced by GoAstV infection is unclear.

Apoptosis can be triggered by a variety of cellular signals during virus infection, including the intrinsic pathway mediated by mitochondria and the extrinsic pathway mediated by death ligands, which further contribute to cell death and tissue lesions (Danthi, 2016). The intrinsic pathway requires permeabilization of the mitochondrial membrane, which is mainly controlled by B-cell lymphoma 2 (Bcl-2) family members, such as proapoptotic effector molecules (Bax and Bak) and antiapoptotic proteins (Bcl-2 and Bcl-xL) (Nagata, 2018). The activation of Bax and Bak subsequently stimulates the release of cytochrome *c* (Cyt *c*) from mitochondria, which further induces the activation of caspase 9 and caspase 3 and leads to apoptosis (Zhou et al., 2017). Many viruses, including influenza A virus, rabies virus and porcine reproductive and respiratory syndrome virus, can induce apoptosis (Lee et al., 2018), but whether GoAstV induces kidney apoptosis in infected goslings has not been reported.

As the first line of host defense, the innate immune system detects and responds to pathogen infection via pattern recognition receptors (PRRs), which recognize conserved molecular structures known as pathogen-associated molecular patterns (PAMPs) that are essential for the life cycle of the pathogen (Kawai and Akira, 2011). Among them, retinoic acid-inducible gene-1 (RIG-I) and melanoma differentiation-associated gene 5 (MDA5), which recognize RNA virus nucleic acids and interact with mitochondrial antiviral signaling (MAVS) (Gurtler and Bowie, 2013). Subsequent activation of downstream adapter proteins, including TANK-binding kinase 1 (TBK1), IκB kinase alpha/beta (IKKα/β), interferon regulatory factor 7 (IRF7) and nuclear factor kappa-B (NF-κB), ultimately induces the transcription of type I interferons and proinflammatory cytokines (Wu and Chen, 2014). NLRP3 is another important PRR belongs to the NOD-like receptor that can be activated by multiple agonists, including PAMPs (such as viral RNAs, microbial toxins and bacterial surface components) and danger-associated molecular patterns (DAMPs) (Huang et al., 2021). Once the NLRP3 inflammasome is activated, it induces pro-caspase-1 cleavage and activation, which results in the maturation and release of proinflammatory cytokines, such as IL-β and IL-18, and further induces inflammatory cell death and inflammatory lesions (Xu and Nunez, 2023).

In the present study, the apoptosis and inflammatory and innate immune responses of goose embryonic kidney (GEK) cells induced by GoAstV infection were investigated to determine the molecular mechanism of pathological lesions in the kidney in gout goslings induced by GoAstV infection as well as the pathogenesis of GoAstV.

## 2 Materials and methods

### 2.1 Viruses and cells

The GoAstV strain GoAstV/Guangdong/QY1/2019 (GoAstV-QY1) (GenBank accession no. ON049473) was isolated from goslings with gout in Guangdong Province, China, following serial passage in goose embryos as described previously (Fu et al., 2022). Primary goose embryo kidney (GEK) cells were prepared from 9-day-old embryonated goose eggs as described previously (Chu et al., 2022). In briefly, the kidney tissues collected from goose embryos were cut into pieces and digested using 0.1% collagenase I (Beyotime, Shanghai, China) for 30 min, and subsequently terminated using Dulbecco’s modified Eagle medium/Nutrient mixture F-12 (DMEM/F12, Gibco) with 10% fetal bovine serum (FBS, Gibco). Then, the solution was passed through 100 μm cell sieves and the suspension was centrifuged at 1,500 rpm for 6 min, and the precipitate were resuspended in DMEM/F12 and centrifuged three times at 300 rpm for 5 min to remove red blood cells. Finally, the segments were resuspended and cultured in in DMEM/F12 supplemented with 10% FBS at 37°C with 5% CO_2_ to obtain GEK cells.

### 2.2 GoAstV infection in GEK cells

Primary GEK cells were cultured in 6-well cell culture plates with DMEM/F12 supplemented with 10% FBS, penicillin (100 U/mL), and streptomycin (100 μg/mL) at 37°C with 5% CO_2_. Subsequently, the GEK cells were infected with GoAstV-QY1 at a multiplicity of infection (MOI) of 0.1 when the cells reached 80% confluence. At 2 h postinfection (hpi), the supernatant of the infected cells was discarded, and DMEM/F12 supplemented with 1 μg/ml TPCK-typsin was added to the cells. The supernatants from the infected cells were harvested every 12 h until 72 hpi, and the virus titer was determined by TCID_50_ as previously described (Ren et al., 2020). In brief, the supernatants were subjected to 10-fold serial dilutions in DMEM/F12 supplemented with 1 μg/ml TPCK-typsin and diluted to a concentration of 1.0 ×10^-8^. Aliquots (200 μL) of the supernatants at each concentration were added to each well of 96-well plates in four replicates, and the infected GEK cells were cultured for 72 hours. At 72 hpi, the infected GEK cells were fixed and analyzed by indirect immunofluorescence assay (IFA) for the detection of the virus, and viral titers were calculated using the Reed-Muench method.

### 2.3 Indirect immunofluorescence assay

GEK cells infected with GoAstV-QY1 for 48 hours were fixed with 4% paraformaldehyde for 5 minutes. After washing with phosphate-buffered saline (PBS) three times, the cells were overlaid with PBS containing 5% w/v bovine serum albumin and incubated for 30 min. Mouse antiserum against the GoAstV capsid protein was used as the primary antibody at a 1:200 dilution in PBS and incubated at 37°C for 1 h. Subsequently, the cells were washed with PBS three times and incubated with fluorescein isothiocyanate (FITC)-conjugated goat anti-mouse IgG (Abcam, USA) as a secondary antibody. The nucleus were then stained with DAPI for 5 min after washing with PBS three times. Finally, the stained cells were examined under a fluorescence microscope.

### 2.4 RNA extraction and quantitative real-time PCR

Total RNA was extracted from GEK cells using TRIzol reagent (Thermo Fisher, USA), and cDNA was synthesized using a PrimeScript™ 1st Strand cDNA Synthesis Kit (Takara, Dalian) according to the manufacturer’s protocol. RT−qPCR was subsequently performed in an ABI 7500 real-time PCR system (Applied Biosystems, USA) with 2× RealStar Green Fast Mixture with ROX II (GenStar, Beijing, China) in a 20 μL reaction system according to the manufacturer’s protocol. The genes tested and the primers used are listed in **Table 1**. The mRNA expression levels of the target genes were normalized to that of β-actin, and the changes in gene expression were calculated using the threshold cycle (2^-△△CT^) method.

**Table 1:** Primers used for real-time PCR in this study.

Tagate gene	Primer sequence (5′- 3′)	GenBank accession no.
RIG-1	F: AGCACCTGACAGCCAAAT	HQ829831
	R: AGTGCGAGTCTGTGGGTT	
MDA5	F: TGCTGTAGTGGAGGATTTG	JX976550
	R: CTGCTCTGTCCCAGGTTT	
MAVS	F: GCCACATCCTGAGGAACAT	XM013182243
	R: GGTATGAAGTTCGTCCCTGTC	
IRF7	F: CACCCGCCTGAAGAAGT	MG707077
	R: GCCCGAAGCAGAGGAA	
NF-κB	F: GCCCAATGCCTCCAACTTAAA	M86930
	R: ATATCATCTTTCTGAACCTTGTCAC	
IL-1β	F: TCCGCCAGCCGCAAAGTG	JF505290
	R: CGCTCATCACGCAGGACA	
IL-6	F: AGATGGTGATAAATCCTGATG	JQ728554
	R: CGGTTTTCTCCATAAATGAAGT	
IL-8	F: ATGAACGGCAAACTTGGGGCT	AB213393
	R: GCCAGAATTGCCTTTACGATCA	
TNF-α	F: TGTGTATGTGCAGCAACCCGTAGT	AY765397
	R: GGCATTGCAATTTGGACAGAAGT	
Bax	F: GGACGAGCTGGACAGCAACG	KY788660
	R: AGGCGGCAGGCGAAGTAGA	
Bcl-2	F: TGACCGAGTACCTGAACCG	XM013187395
	R: GCTCCCACCAGAACCAAA	
Caspase-1	F: CCAGGCAGAAGTGGATGAGG	MG463109
	R: GCACCAGGAGGCTCTGCAT	
Caspase-3	F: ACGTGTCAGAAAATACCTGT	KF787121
	R: AGTTCAAGTTTCCGTGCAT	
Caspase-9	F: GCTGTTTCAACTTCCTCCGTA	XM013187987
	R: ATAGCTTTCGTCCGGCCAT	
NLRP3	F: CTCCTTGCGTGCTCTAAGACC	OP331217
	R: CTTGTGCTTCCAGATGCCGTC	
β-actin	F: TGACGCAGATCATGTTTGAGA	M26111
	R: GCAGAGCGTAGCCCTCATAG	

### 2.5 Western blot analysis

GEK cells infected with GoAstV were lysed with radioimmunoprecipitation assay (RIPA) buffer supplemented with protease inhibitors (Beyotime, Shanghai, China). The protein concentration was determined by the BCA method using a Pierce BCA protein assay kit (Beyotime, Shanghai, China). The lysates were loaded with 6× denaturing sample buffer, separated by 10% SDS−PAGE and transferred to a PVDF membrane (Thermo Fisher Scientific, Waltham, MA). The proteins were separated using PBST containing 5% skimmed milk and blocked, and the membranes were incubated with the indicated primary antibodies overnight at 4°C. The membranes were washed three times with PBST and then incubated with horseradish peroxidase (HRP)-conjugated secondary antibodies for 1 h at 37°C (Beyotime, Shanghai, China). The membranes were washed three times with PBST and then visualized via enhanced chemiluminescence reagents (Bio-Rad, California, USA). The intensity of the bands was analyzed using the ImageJ 1.52v image processing program (National Institutes of Health, Bethesda, MA).

### 2.6 Statistical analysis

Statistical analyses were carried out using GraphPad Prism 6 (GraphPad Software, La Jolla, CA). Student’s t test was used to calculate p values, all experiments were conducted in triplicate, and all data are shown as the means ± standard deviation (SD). Significance levels were set as follows: *, p < 0.05; **, p < 0.01; ***, p < 0.001.

## 3 Results

### 3.1 The infection and replication of GoAstV in GEK cells

**Figure 1:**
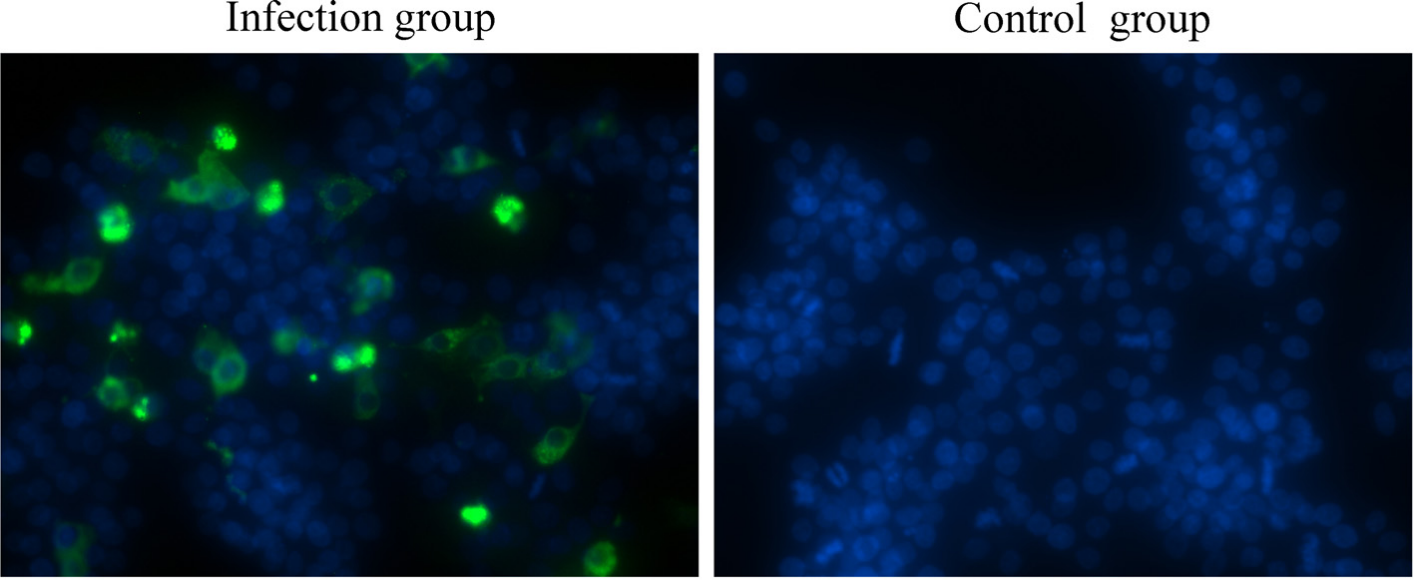
GEK cells infected with GoAstV were identified by indirect immunofluorescence at 48 hours post infection.

One of the major barriers to understanding astrovirus pathogenesis is the lack of suitable cell culture systems (Johnson et al., 2017). In the present study, a GEK cell infection model for GoAstV was established, and the results showed that GoAstV can effectively infect and proliferate in GEK cells (**Figure 1**), and the viral titer can reach 3.58×10^5^ TCID_50_/mL at 72 hours post infection.

### 3.2 GoAstV infection induces apoptosis in GEK cells

**Figure 2:**
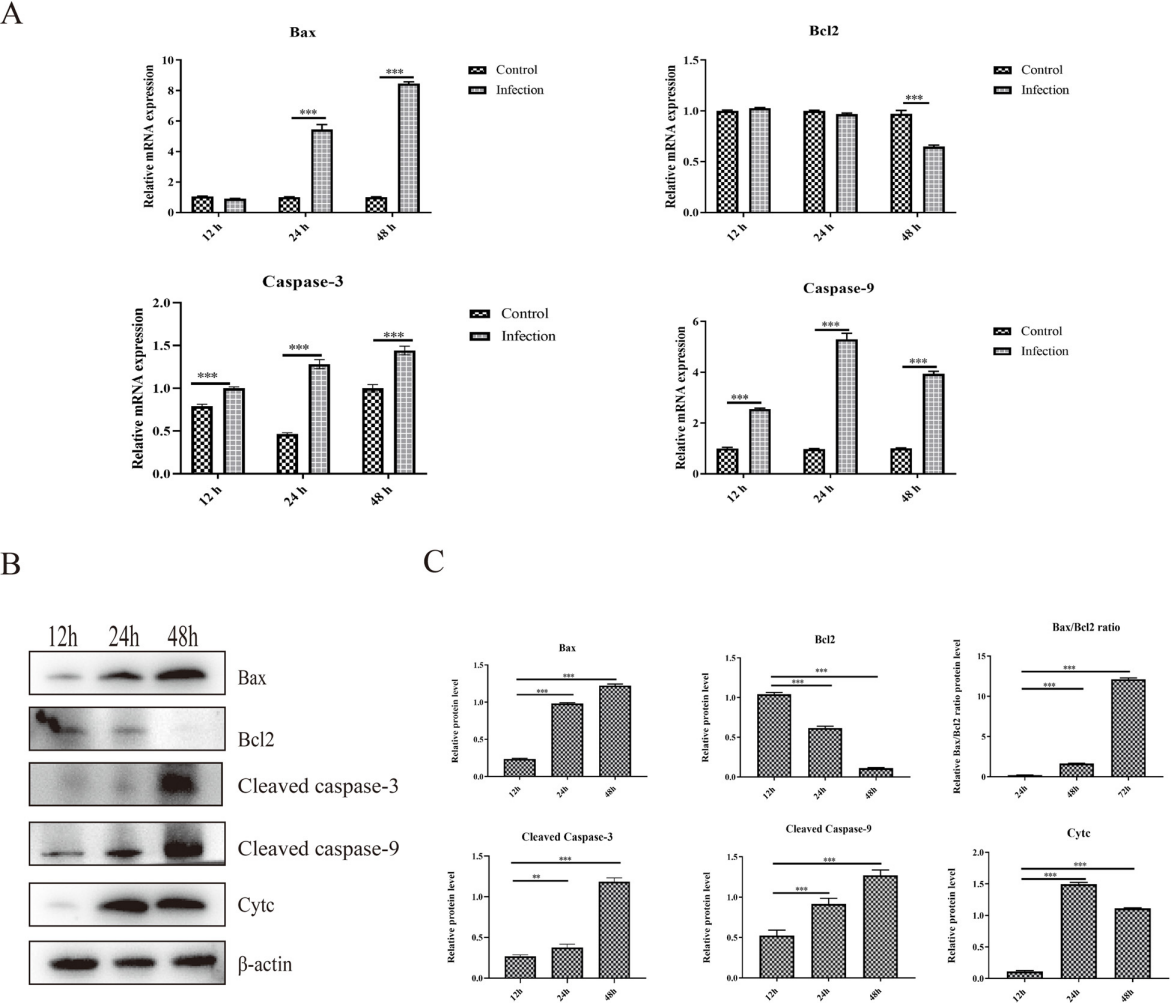
Apoptosis induced by GoAstV infection in GEK cells. **(A)** The mRNA expression levels of Bax, Bcl-2, Caspase-3 and Caspase-9 at 12 hpi, 24 hpi and 48 hpi. **(B)** The protein expression levels of Bax, Bcl-2, cleaved caspase-3, cleaved caspase-9 and cytochrome *c* (Cyt *c*), which are responsible for mitochondrial apoptosis, in GEK cells infected with GoAstV at 12 hpi, 24 hpi and 48 hpi. **(C)** The histogram summarizes the protein expression levels. **, p < 0.01; ***, p < 0.001.

Apoptosis is a type of programmed cell death that can be induced by viral infection, which can further restrict viral replication and contribute to tissue lesions. The mRNA and protein expression levels of critical proteins responsible for apoptosis were determined, and the results showed that the mRNA expression levels of proapoptotic proteins, including Bax, caspase-3 and caspase-9, were significantly greater in the infection group than that in the control group at 24 hpi and 48 hpi (**Figure 2A**). Moreover, the mRNA expression level of the antiapoptotic protein Bcl-2 was significantly down-regulated about one-third at 48 hpi than that in the control group (**Figure 2A**). In addition, the protein expression levels of Bax, cleaved caspase-3, cleaved caspase-9 and Cytc increased significantly from 12 hpi to 48 hpi in the infection group (**Figures 2B, C**). However, the protein expression level of the critical antiapoptotic protein Bcl-2 decreased significantly from 12 hpi to 48 hpi in the infection group, which resulted in an increase in the ratio of Bax to Bcl-2. (**Figures 2B, C**). These results showed that GoAstV infection could induce apoptosis in GEK cells via the mitochondrial pathway.

### 3.3 Activation of the RIG-I/MDA5 pathway and inflammatory cytokine expression induced by GoAstV infection

**Figure 3:**
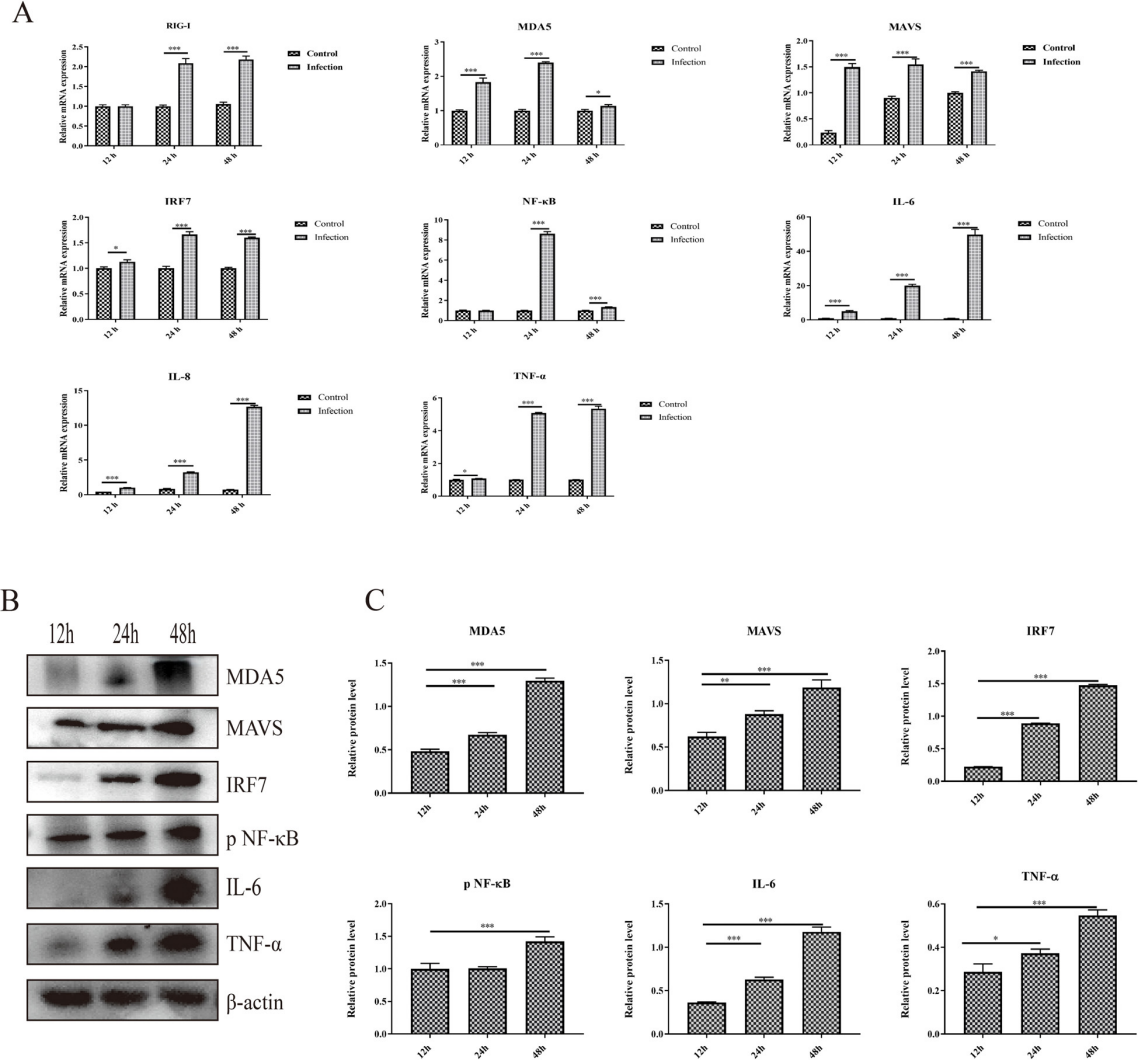
The activation of the RIG-I/MDA5 pathway and inflammatory cytokine expression induced by GoAstV infection. **(A)** The mRNA expression levels of genes involved in the RIG-I/MDA5 pathway (RIG-I, MDA5, MAVS, IRF7 and NF-κB) and inflammatory cytokines (IL-6, IL-8 and TNF-α) in GEK cells infected with GoAstV at 12 hpi, 24 hpi and 48 hpi. **(B)** The protein expression levels of genes involved in the RIG-I/MDA5 pathway (MDA5, MAVS, IRF7 and pNF-κB) and inflammatory cytokines (IL-6 and TNF-α) in GEK cells infected with GoAstV at 12 hpi, 24 hpi and 48 hpi. **(C)** The histogram summarizes the protein expression levels. *, p < 0.05; **, p < 0.01; ***, p < 0.001.

The activation of the RIG-I/MDA5 pathway plays an important role in the production of type I interferons and inflammatory cytokines, and whether GoAstV infection induces RIG-I/MDA5 pathway activation and further induces the excessive expression of proinflammatory cytokines was also determined. As shown in **Figure 3A**, the mRNA expression levels of RIG-I, MDA5 and key adapter proteins, including MAVS, IRF7 and NF-κB, were significantly upregulated in the infection group at 24 hpi and 48 hpi. Moreover, the protein expression levels of key adapter proteins, including MDA5, MAVS, IRF7 and phosphorylated NF-κB, increased from 12 hpi to 48 hpi (**Figures 3B, C**), which is consistent with the gene expression analysis.

In addition, the mRNA expression levels of proinflammatory cytokines downstream of the RIG-I/MDA5 pathway, including IL-6, IL-8 and TNF-α, were also significantly upregulated in the infection group than that in the control group at 12 hpi, 24 hpi and 48 hpi, respectively (**Figure 3A**). Moreover, the protein expression levels of proinflammatory cytokines (IL-6 and TNF-α) increased from 12 hpi to 48 hpi (**Figures 3B, C**). These results indicate that infection with GoAstV can activate the RIG-I/MDA5 pathway and induce the release of proinflammatory cytokines downstream, which further leads to inflammatory lesions in the kidney.

### 3.4 Activation of NLRP3 induced by GoAstV infection

**Figure 4:**
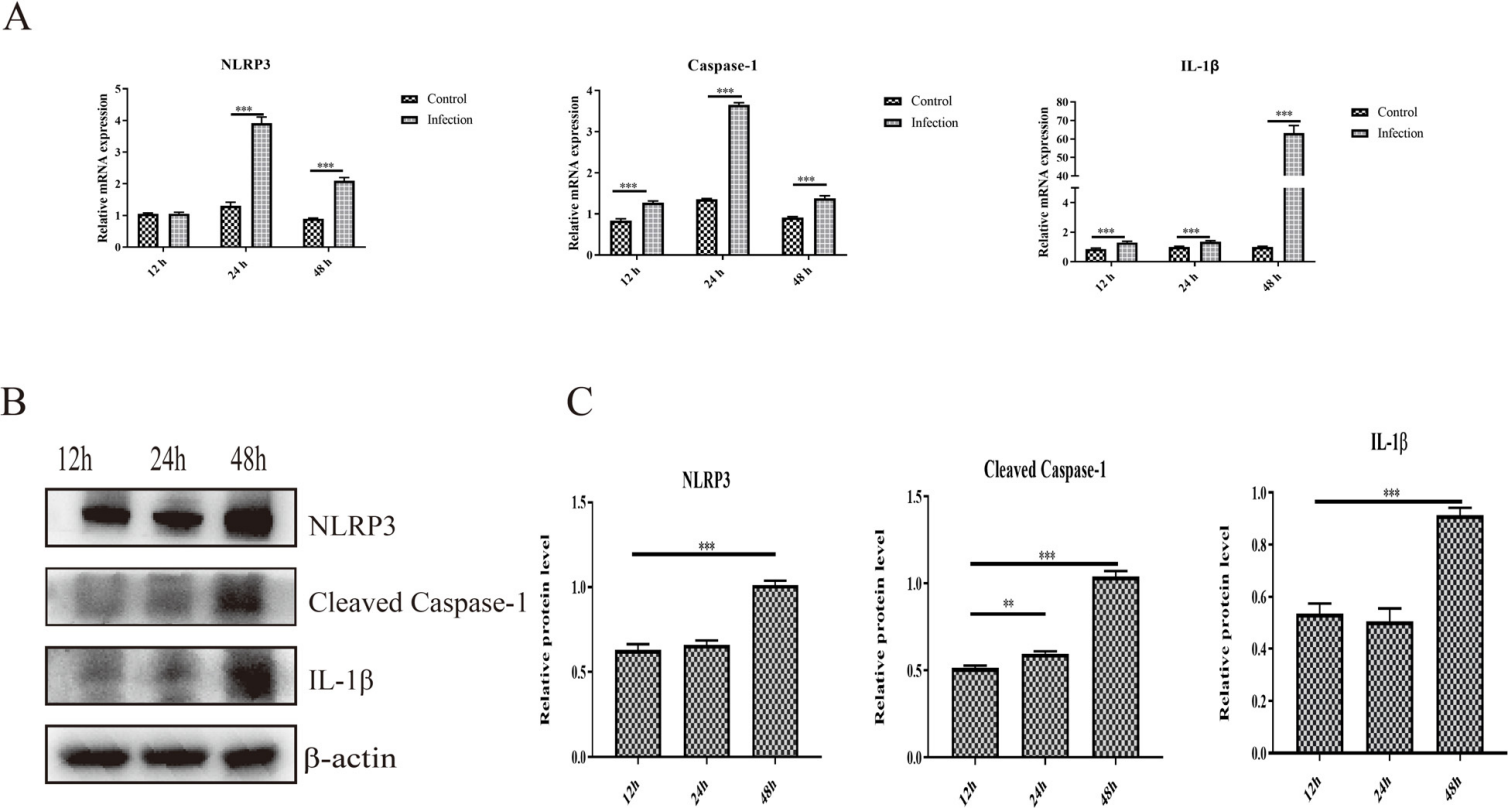
Activation of NLRP3 induced by GoAstV infection in GEK cells. **(A)** The mRNA expression levels of NLRP3, Caspase-1 and IL-1β in GEK cells infected with GoAstV at 12 hpi, 24 hpi and 48 hpi. **(B)** The protein expression levels of NLRP3, cleaved caspase-1 and IL-1β in GEK cells infected with GoAstV at 12 hpi, 24 hpi and 48 hpi. **(C)** The histogram summarizes the protein expression levels. **, p < 0.01; ***, p < 0.001.

The activation of NLRP3 is responsible for the cleavage of caspase-1 and the maturation and release of proinflammatory cytokines, which further induces inflammatory cell recruitment and inflammatory lesions. In the present study, we found that infection with GoAstV activated the NLRP3 pathway, and the mRNA expression levels of NLRP3, cleaved caspase-1 and IL-1β were significantly upregulated in the infection group at 24 hpi and 48 hpi compared to those in the control group (**Figure 4A**). In addition, the protein expression levels of NLRP3, cleaved caspase-1 and IL-1β increased significantly from 12 hpi to 48 hpi in GEK cells infected with GoAstV (**Figures 4B, C**), which indicates that GoAstV infection induces the activation of the NLRP3 pathway in GEK cells and further induces inflammatory lesions in the kidney upon infection with GoAstV.

## 4 Discussion

Gout outbreaks in goslings caused by the novel GoAstV have resulted in considerable economic losses in the goose industry in China (Niu et al., 2018). However, the underlying molecular pathogenic mechanisms of gout caused by GoAstV and the interaction of GoAstV with the host are unclear, mainly because of the lack of suitable cell culture systems. It is well known that the isolation and culture of astroviruses *in vitro* is difficult, and the infection and replication of astroviruses generally show self-limitation in cell culture (Johnson et al., 2017; Ren et al., 2020). Previous studies have reported that LMH cells can be used for GoAstV isolation and replication, but self-limiting infection in LMH cells has been reported (Ren et al., 2020; Zhang et al., 2018b). In the present study, a GoAstV infection model in GEK cells was established, and GoAstV could effectively infect and proliferate in GEK cells, which is helpful for further understanding the pathogenic mechanisms and interactions between GoAstV and host cells.

Wu et al. reported that GoAstV infection in goslings can cause lesions on the liver and kidney and further increase the activity and expression of enzymes related to uric acid production in the liver and decrease renal excretion, which leads to hyperuricemia and gout formation (Wu et al., 2020). The kidney is considered to be the main target tissue for GoAstV infection, and it shows severe lesion and histopathological changes (Zhang et al., 2018a). However, the molecular mechanism of pathological lesions in the kidney induced by GoAstV and the interaction between GoAstV and the host are unclear. Previous studies have reported that human astrovirus infection can induce apoptosis in CaCo-2 cells and is necessary for efficient human astrovirus replication and particle maturation (Guix et al., 2004). In addition, Ding et al. reported that GoAstV could induce apoptosis in splenic lymphocytes in geese at 3 and 7 days post infection via the death receptor-mediated extrinsic apoptosis pathway (Ding et al., 2021). Here, we found that GoAstV infection could induce apoptosis in GEK cells via the intrinsic pathway, which is characterized by the upregulation of proapoptotic proteins (Bax, caspase-3 and caspase-9) and the downregulation of antiapoptotic proteins (Bcl-2). Apoptosis induced by viral infection is considered to be an antiviral pathway activated by the host to limit virus replication (Upton and Chan, 2014). In addition, studies in some systems have demonstrated that apoptosis also contributes to viral disease by causing tissue injury (Danthi, 2016; DeBiasi et al., 2004). Thus, apoptosis induced by GoAstV infection may be an important factor for kidney lesions, and the underlying mechanism needs to be further investigated.

Innate immunity is the first line of defense against pathogen invasion and initiates host antimicrobial responses, such as the production of type I interferons and proinflammatory cytokines (Gurtler and Bowie, 2013). A previous study reported that porcine astrovirus infection induces the production of IFN-β via the RIG-I and MDA5 signaling pathways and further inhibits viral replication (Dong et al., 2023). Wu et al. reported that GoAstV induced the activation of PRRs (RIG-I, MDA5 and TLR3) and key adaptor molecules (MyD88, MAVS and IRF7) in the spleen and kidney in infected geese, as well as high expression of proinflammatory cytokines, including IL-1β and IL-8 (Wu et al., 2021). In the present study, downstream activation of the RIG-I/MDA5 pathway and upregulation of proinflammatory cytokines (IL-1β, IL-6, IL-8 and TNF-α) were observed in GoAstV-infected GEK cells, which is consistent with previous studies. The activation of the RIG-I/MDA5 pathway is helpful for the production of IFN-β and antiviral responses to limit the replication of GoAstV in host cells. However, the overexpression of proinflammatory cytokines is a critical factor for inflammatory lesions and inflammatory disease (Kany et al., 2019). The upregulation of proinflammatory cytokines in GoAstV-infected GEK cells may play an important role in kidney lesions, which may decrease renal excretion of uric acid and further induce hyperuricemia and gout formation (Wu et al., 2020).

NLRP3 is a primary sensor of sterile inflammatory signals and a key regulator underlying chronic disease driven by inflammation (Wang and Hauenstein, 2020). The NLRP3 inflammasome is associated with inflammatory responses triggered by viral infection; for example, influenza virus infection can activate NLRP3 and subsequently stimulate caspase-1 activation and the production of IL-1β and IL-18, resulting in increased neutrophil and lymphocyte recruitment to the respiratory tract in infected mice (Allen et al., 2009). Here, we found that GoAstV infection could also induce the activation of the NLRP3 pathway and stimulate the increased production of the proinflammatory cytokine IL-1β in GEK cells. Combined with severe inflammatory lesions and histopathological changes in the kidneys of infected geese (Zhang et al., 2018a), these findings suggest that activation of the NLRP3 pathway has a potential relationship with inflammatory lesions in the kidney, but further investigations are needed.

In summary, the present study investigated the apoptosis and inflammatory and innate immune responses induced by GoAstV in GEK cells, which is helpful for understanding the molecular mechanism of pathological lesions in the kidney in gout gosling as well as the pathogenesis of GoAstV.

## Data Availability

The original contributions presented in the study are included in the article/supplementary material. Further inquiries can be directed to the corresponding author.
